# Focus on autism and other neurodevelopmental disorders

**DOI:** 10.1111/gbb.12789

**Published:** 2021-12-10

**Authors:** Andrey E. Ryabinin

**Affiliations:** ^1^ Department of Behavioral Neuroscience Oregon Health & Science University Portland Oregon USA

**Keywords:** autism, DISC1, Prader‐Willi syndrome, neurodevelopment, Williams Syndrome

Understanding mechanisms underlying mental and neural disorders should be difficult by definition because of the complexity of mechanisms involved in regulation of behavior. This complexity is multiplied when the disorder involves social phenotypes because behavior, in this case, involves more than one organism. The difficulty of understanding mental and neural disorders is further multiplied if the disorder involves developmental changes.

Therefore, deciphering mechanisms of autism and other neurodevelopmental disorders sometimes appears unsurmountable. Experiments in this research area have to provide balanced tests across potential pathological factors, genetics, sex/gender, and ages of the studied subject. Moreover, effects of these factors have to be analyzed across more than one of phenotype in an attempt to distinguish which of the phenotypes reflect the potential main culprit in the development of the disorder. Experiments analyzing effects of so many factors and across multiple behavioral phenotypes are difficult to execute. As a result, knowledge about neurodevelopmental disorders is accumulated in a mosaic fashion: A study might analyze many factors, but not across multiple ages, or focus on only one measure of behavior, or, in contrast, analyze multiple behaviors, but not take into account genetics or sex of the subjects. Because of difficulty of interpreting such mosaic evidence, we especially value studies that managed to achieve balanced analysis of more than one factor and more than one behavioral measure in the same set of experiments. This special issue of *Genes, Brain, and Behavior*, focusing on rodent models of autism and other developmental disorders, contains several of such studies. Some of them produced unexpected results.

Subjects with autism spectrum disorders (ASD) are characterized by challenges with social communication and interactions as well as restricted or repetitive behaviors.^1^ However, additional phenotypes are frequently associated with this disorder. For example, subjects with ASD frequently report abnormal somatosensory responses. In agreement, studies modeling ASD in rodents have reported altered behavior in nociceptive tests.^2^, ^3^ However, this evidence varied, depending on the model of ASD and nociceptive test used. In their thorough study published in this issue, Martin and colleagues analyzed male mice of two genetic models of ASD in an extensive set of tests of nociceptive behaviors and emotional contagion of pain.^4^ They found that the mosaic evidence for the varied directions of altered nociception in ASD can be replicated within a single study and even within a single model of ASD. Thus, BTBR mice, an idiopathic model of ASD, showed decreased sensitivity to mechanical, heat, and peripherally applied chemical stimuli but increased sensitivity to visceral sensation in acute tests. On the other hand, Fmr1‐KO mice, a monogenic model of ASD, showed increased sensitivity to mechanical and decreased sensitivity to chemical stimuli, but normal sensitivity to thermal or visceral stimuli in acute tests. Differences in nociception were also observed between and within these genetic models in tests of chronic pain. In contrast to these varied responses in tests of nociception, both BTBR and Fmr1‐KO mice showed clear deficits in motivation for social interaction and in two tests of emotional contagion, in agreement with being the models of ASD. While these studies suggest that abnormal somatic sensation is an unlikely culprit for ASD, they indicate that observations of altered pain sensation in ASD are not a consequence of mosaic studies, but indeed reflect the complex nature of symptoms accompanying this disorder.

ASD is also frequently associated with attentional abnormalities. In fact, there is high co‐morbidity between ASD and the attention‐deficit hyperactivity disorder.^5^ In the present issue, Burrows and colleagues assessed attention in male NL^R451C^ mice.^6^ NL^R451C^ mice carry an arginine‐to‐cysteine residue substitution in neuroligin‐3, an X‐linked polymorphism associated with this disorder. Attention was assessed in two comprehensive touchscreen tests: The 5‐choice serial reaction time task and the rodent continued performance test. These two different tasks either require the mice to attend to the entirety of touchscreen, and thus assess visuo‐spatial attention, or test attention to a single target stimulus while inhibiting distractor stimuli, respectively. Thus, while assessing different types of attentional processes, the two tasks offer great translational capacity due to implementation of touch screen technology. The authors observed slower response times and quicker reward collection, enhanced ability to attend to stimuli when the task load was low, as well as less responses to distractor stimuli and lower false alarm rates. These findings indicate attentional deficits are not inherent in ASD and, in fact, agree with a few studies demonstrating enhanced attention in some ASD patients.^7^, ^8^

Prader–Willi syndrome (PWS) is a genetic neurodevelopmental disorder resulting from a loss of paternally inherited genes on Chromosome 15 associated with hypotonia, abnormalities in feeding and social behaviors, as well as cognitive deficits. Magel 2 is one of the candidate genes for PWS, and humans with loss‐of‐function polymorphisms in Magel 2 show symptoms of PWS and ASD.^9^ In this issue, Bosque Ortiz and colleagues investigated the development of vocal communication in male and female Magel2‐deficient mouse pups across 4 postnatal ages.^10^ Analyzing separation‐induced vocalizations, they found lower rates of such vocalizations and altered spectral features in Magel2 mutants. Detailed analyses of the vocalizations showed that the vocal repertoire of these mutant pups at postnatal day 8 was similar to that of PN10 and 12 of wildtype pups, paradoxically suggesting faster development to the more mature phenotype. Since appropriate separation‐induced vocalizations are required for adequate feeding and growth of young pups, this deficiency would potentially affect their future development resulting in abnormalities across several cognitive and affective domains.

The value of analyzing behaviors at several developmental ages, albeit during periadolescence, is also demonstrated in a study by Glenn and colleagues.^11^ They have focused on the developmental aspects of behavioral differences in male and female DISC1 knockout rats. By incorporating an early cross‐fostering scheme, they were able to take into account potential differences in rearing conditions between mutant and wildtype animals. Serial tests evaluating exploratory/anxiety‐like behaviors, social, and nonsocial exploration and prepulse inhibition were performed between postnatal days 21 and 36. DISC1 encodes a scaffolding protein involved in neurodevelopment and its abnormal activity is thought to contribute to development of schizophrenia.^12^ While men appear to be at a higher risk for the development of this mental disorder, women tend to show slightly different types of symptoms.^13^, ^14^ Interestingly, although phenotypes tested at later ages in this study do not show robust male–female differences, the authors detected a substantially higher exploratory/locomotor activity in female, but not male, DISC1 knockouts. Sex differences in the development of mental disorders need further exploration.

The ultimate goal of animal models of neurodevelopmental disorders is to suggest treatments that could be tested in clinical trials. In this issue, Nygaard and colleagues investigated one such potential treatment in a mouse model of the Williams Syndrome (WS).^15^ WS results from a hemizygous deletion of 26–28 genes on Chromosome 7. Symptoms of WS include craniofacial dysmorphology, connective and cardiac tissue abnormalities, complex cognitive, sensory processing disabilities, nonsocial anxiety, but surprisingly high sociability and emotional sensitivity to music.^16^ Abnormalities in the oxytocin system, which is well known to play one of the leading roles in regulation of social behaviors, are suspected to contribute to WS.^17^ In the present study, the authors investigated the complete deletion (CD) hemizygous mouse mutants, deficient in the genes corresponding to WS, in contextual and cued fear conditioning, well‐established tests of associate memory. They demonstrate that CD mice have a profound deficit in both types of these associative tests. The authors then tested the potential contribution of the oxytocin system to the observed phenotype. They found no significant differences in measures of oxytocin activity between CD and wildtype mice, and central administration of the oxytocin receptor antagonist did not affect fear conditioning in either genotype of test subjects. While attempting to manipulate the oxytocin system appears feasible in clinical settings, the negative findings of this study call for more sophisticated approaches to treatment of neurodevelopmental disorders.

Exciting developments in the possibility of such sophisticated treatments are presented in two other articles published in this issue.^18^, ^19^ There, two groups of researchers investigate genetic therapy approaches for the treatment of Rett Syndrome. These articles are previewed by a separate Editorial.^20^

Overall, basic science studies using models of neurodevelopmental disorders identify at times surprising aspects of investigated phenotypes. The complexity of mechanisms is expected to be especially striking when researchers attempt to tackle social behaviors. Therefore, a better understanding of natural (as opposed to pathology‐related) species‐specific social behaviors is needed. This topic will be explored in the next issue of *Genes, Brain, and Behavior*.

## Data Availability

Data sharing is not applicable to this article as no new data were created or analyzed in this study.
